# Escalation and de-escalation of the radiology response to COVID-19 in a tertiary hospital in South London: The King’s College Hospital experience

**DOI:** 10.1259/bjr.20201034

**Published:** 2020-11-23

**Authors:** Andreas Panayiotou, Vasileios Rafailidis, Thomas Puttick, Keshthra Satchithananda, Adam Gray, Paul S Sidhu

**Affiliations:** 1 Department of Radiology, King's College Hospital, London, UK; 2 Chelsea and Westminster Hospital, NHS Foundation Trust, London, UK

## Abstract

The pandemic of COVID-19 presented an enormous challenge to the medical world in terms of diagnosis, treatment and health-care management as well as service organisation and provision. This novel virus and its spread affected every aspect of modern medical practice, ranging from investigating transmission of this new pathogen, antigen testing of symptomatic patients, imaging, assessing different treatment regimens and the production of a new vaccine. Imaging played a crucial role in the diagnosis of COVID-19-related lung disease, with plain radiography and CT being the main diagnostic modalities, with ultrasound a useful bedside imaging tool. The accurate and early diagnosis of the disease was not the only issue faced by Radiology Departments across the world; prevention of nosocomial infection, creating capacity with elective imaging suspension, management and protection of the workforce being few of the numerous challenges. The purpose of this manuscript is to present the steps that the Radiology Department of a large urban tertiary facility with a local vulnerable population, undertook to adapt the imaging service and structure, both initially escalating and then de-escalating a response to the COVID-19 pandemic. A step-by-step management strategy, effective and sustained staff deployment, imaging management are presented and discussed, to provide a guide for managing a major incident in a radiology department.

## Introduction

King’s College Hospital (KCH) is one the largest and busiest teaching hospitals covering multiple boroughs in the south of London, representing a large urban tertiary facility. The Boroughs of Lambeth and Southwark are located in South London and have a vulnerable local population that are below the national average for numerous public health indicators such as homelessness, deprivation, violent crime, obesity and sexually transmitted diseases. The hospital operates from 3 acute sites with more than 1300 beds, including 144 intensive care unit beds.^1^ In addition to providing comprehensive health care for the local population, as part of the National Health Service (NHS) of the United Kingdom, the hospital provides regional and national services for liver disease (both adult and paediatric) with the largest liver transplantation programme in Europe, a Level 1 trauma facility, comprehensive haematological service including bone marrow transplantation, a regional neurology and neurosurgical facility, a regional neonatal intensive care unit, cardiovascular and renal services to a large population base. The imaging department is a large facility divided across sites, concentrated at the KCH Denmark Hill, Princess Royal University Hospital (PRUH) and the Orpington Hospital sites, with imaging services geared to service the requirements of the tertiary services and the local health-care needs. Intensive care services are concentrated at the KCH Denmark Hill site with 79 adult, 16 paediatric and 24 neonatal critical care beds. The remaining critical care beds are based in the PRUH. Specialist imaging services are concentrated at the Denmark Hill site and include dedicated neuroimaging, stroke imaging and treatment, trauma, breast imaging, nuclear medicine, and specialist ultrasound. Dedicated interventional radiology is provided for neurological, hepatobiliary, vascular, trauma and paediatrics. The hospital group records over 500 000 imaging episodes each year. The PRUH is a district general hospital with emergency department, medicine, surgery and outpatients. The Orpington hospital is a district general hospital with surgery and outpatients. Each site has their own dedicated diagnostic and intervention services.

The onset and rapid development of the pandemic was anticipated and preparations instituted at an early stage. The first patient diagnosed with Coronavirus 2019 (COVID-19) infection at KCH was at the end of February 2020, with WHO categorising the infection as a pandemic on 11 March 2020. Thereafter, the hospital experienced an unprecedented surge in infections, with the largest number of in-patients in the United Kingdom admitted to a single hospital group. As of early August 2020, the total number of patients admitted to the hospital was just over 3000. De-escalation of measures started in mid-May, by then COVID-19 inpatients and new admissions had significantly subsided.

The main presentation of COVID-19 is a lower respiratory tract infection, with the majority of symptomatic patients requiring imaging with radiography and CT for detection, grading severity and follow-up.^2^ Although Radiology is not deemed a front-line service for immediate patient care, the department had a crucial role in prompt diagnosis, triage and management of patients. Moreover, radiology staff were vulnerable to viral exposure through portable X-ray, ultrasound and interventional procedures. Prevention of cross-infection was of paramount importance, particularly with the known highly contagious nature of the virus. Workforce and operational modifications were required to facilitate the surge of admissions to ensure the safety and wellbeing of staff.

We describe a two-step process of escalation and de-escalation response of a large tertiary hospital in London, focusing on the role of radiologists and allied health-care staff and reconfiguration of the department to provide efficient imaging services, facilitate patient flow and maintain infection control.

### Escalation

#### National and for breast screening recommencement, with all cancellations government response

A nationwide response was led by the government, to prepare the NHS and the country for the COVID-19 pandemic. Implementation for the NHS included: post-ponement of all non-urgent operations; building temporary field hospitals, *i.e*. NHS Nightingale Hospitals, to provide additional critical care capacity for existing NHS hospitals; private hospitals to provide urgent operations and cancer treatment for NHS patients; re-registering of doctors who have recently left the profession; recruiting final-year medical student and nurses into temporary roles.^3^ The government’s response is only briefly outlined as the full extent of the national response to the pandemic is beyond the scope of this article.

The NHS Nightingale London was based in the ExCeL conference centre in East London (nine miles north-east to the KCH Denmark Hill site), providing 500 intensive care beds with the potential of expanding an additional 3500, admitting stable intubated and ventilated COVID-19 patients. The purpose was to allow for intensive care facilities in the existing London hospitals to manage the more unstable patients, in an event where there is an overwhelming patient surge requiring high level care rendering the NHS unable to cope with demand.^4,5^ Doctors volunteered to work at the centre from the whole of London.

### Hospital response (Table 1)

**Table 1.** Summary of issues and steps taken through the escalation phase of the hospital and radiology response to the pandemic

Escalation of the Hospital and Radiology response **to the pandemic**	
Fields	**Steps**
Patient pathways	Restricting visitors Rearrangement of hospital entrance, exit and waiting rooms Cancellation of elective/non-urgent patient appointments Tele-consultations to patients Private independent hospitals used for cancer and non-urgent NHS patient’s surgery
Redeployment	Junior Doctors moved to ICU, medical wards and Emergency Departments
Rota/staff changes	24/7 consultant presence Three groups of consultant/senior trainees and radiographers were grouped covering alternating shifts throughout the day and night Tele-radiology night cover suspended
PPE	Adequate availability Site requiring PPE designation signs Identification of roles/interventions requiring different types of PPE (*e.g.* surgical masks *vs* FFP3) Fit test
Wellness and psychological support	Telephone helplines Creation of hubs where charitable food donations were available
Patient and family liaison service	Setting up telephone service for patients and their families since visits were not allowed.
Radiology site modifications	Dedicated COVID-19 CT, MRI, angiography and X-ray suites designated. Portable imaging modalities (X-ray and ultrasound) opted wherever possible. Dedicated ultrasound machines in every ICU. PACS workstations provided for home reporting.

ICU, Intensive care unit; PACS, Picture archiving and communication system ; PPE, Personal protective equipment.

### Reconfiguration of patient pathways

Post-ponement of non-essential and face-to-face consultations was implemented to reduce the risk of exposure to patients and staff, and redirect non-front-line staff to COVID-19 wards, emergency department and intensive care unit (ICU). Introduction of virtual clinics and telephone consultations for primary health care and outpatients reduced patient and clinician exposure during delivering appropriate care. The emergency department was strictly divided into COVID-19 and non-COVID-19 areas, to stop cross-infection between patients. There was a significant drop in emergency attendances, likely due to lock-down measures and public fear, which allowed the emergency department to focus resources to deal with the pandemic but also treat those clinically urgent non-COVID-19 patients. Access to the hospital was restricted; security administered a single entrance and exit site to monitor access into the hospital, no visitors were allowed, without written permission, all staff had identification checked. Patients and visitors were encouraged to maintain social distancing and to wear a mask at all times. Hand hygiene was enforced at the hospital entrance.

### Workforce

#### Redeployment

Mass redeployment of staff was orchestrated to help the front-line COVID-19 response services that included redeployment of various grades of specialist doctors, nursing and other allied health-care staff from services which were markedly reduced or cancelled. Directives were received from government agencies that a provision for a 24 h continuous consultant presence in the hospital should be implemented. There was a mass redistribution of administrative staff to provide duties such as caring roles on the wards. Management was a top-down approach with redeployment generally being to intensive care or medical ward teams with redeployed staff joining a tailored COVID-19 rota with set responsibilities based on staff experience and clinical need. Consideration of staff absence due to sickness leave (estimated at 25%) was factored into rota preparation. This was used to help decide redeployment numbers in cases of mass absence, to reduce the risk of inadequate staff levels and prevent staff fatigue. When redeployment was deemed clinically urgent there was as little as 24 h’ notice for redeployment, but with rapid inductions and clarification of new role within the hospital. Occupational health assessments for staff, prior to redeployment, identified those who could assume a patient-facing role and those with health risks who should be in a non-patient facing role. National guidelines were followed for those staff deemed as “clinically extremely vulnerable group” due to age and health co-morbidities were advised to work from home where possible.

#### Personal protective equipment

Personal protective equipment (PPE) was sourced promptly and strict guidance on indications for wearing PPE was enforced as per Public Health England guidance,^6^ with appropriate signage in each department. PPE was to be used appropriately to limit wastage and prevent shortages, in contradistinction to using high level PPE protection (FFP3 mask, surgical gown and eye protection) for low risk patients that were not suspected to have COVID-19. The hospital engaged their procurement department to manage the supply chain to ensure sufficient PPE was obtained and distributed to relevant areas with “just in time” stock control. COVID-19 areas were clearly signposted and posters of guidance of the required PPE for access or different types of patient contact (aerosol generating procedures *vs* non-aerosol generating) was clearly displayed in all clinical areas to ensure staff were well-informed and access was restricted to only those who needed to enter. Protective face mask “fit testing” was carried out for all staff across the hospital.

#### Wellness and psychological support

**Figure 1.**
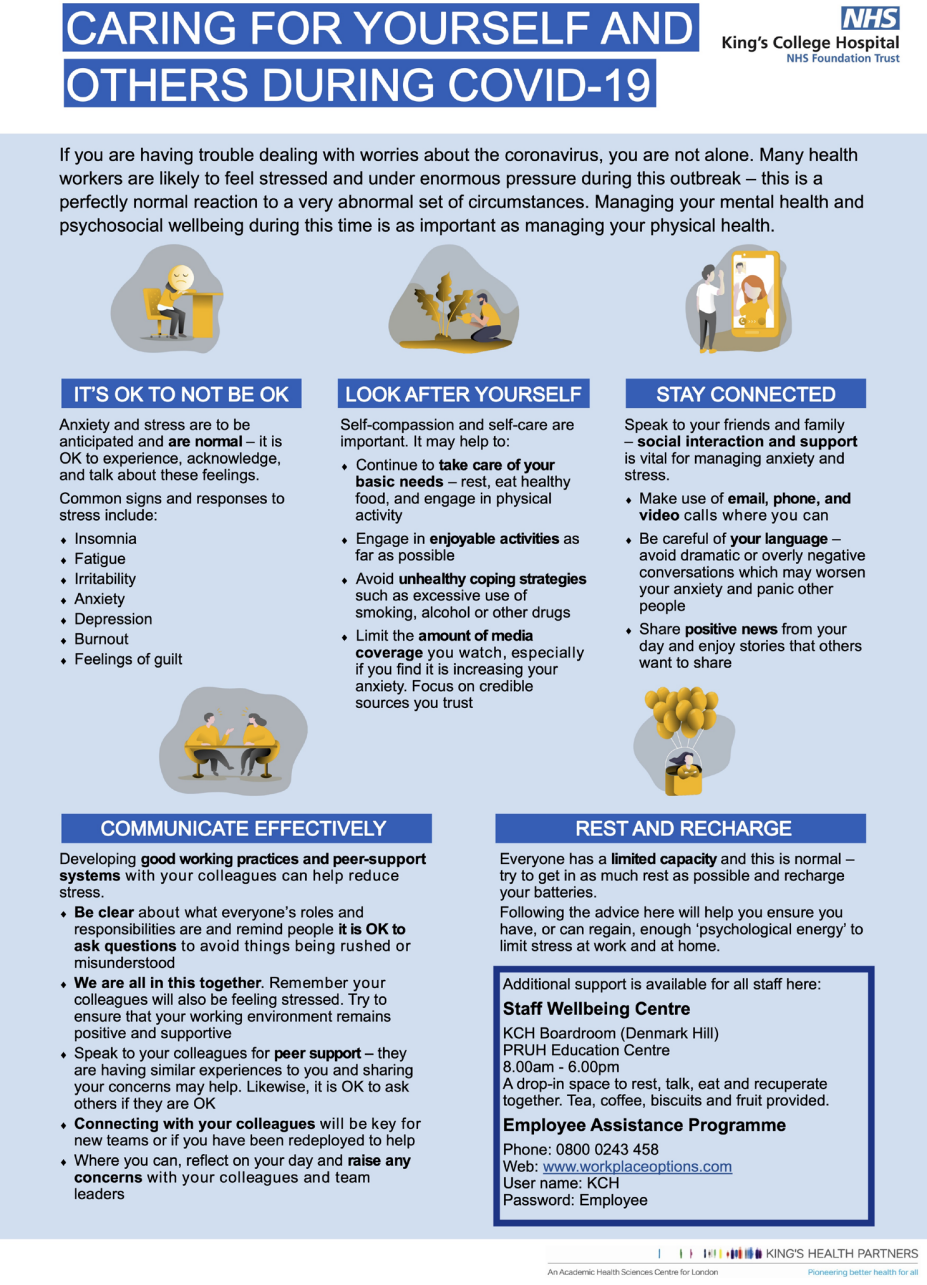
“Caring for yourself and other during COVID-19”. Courtesy of King’s Health Partners.

Well-being hubs were instituted and provided respite for staff and also allowed for psychological support. These allowed space and time for teams to bond and concerns to be discussed with colleagues. There were provisions in place to help with staff transport to the hospital when public transport was limited, this including reimbursements for taxis and car-sharing between staff. Free accommodation was provided for clinical staff who were unable to commute or had vulnerable family members at home. The Southwark and Lambeth boroughs to which the KCH Denmark Hill is located, allowed temporary lifting of parking restrictions locally to allow staff to park and avoid travelling on public transport. Further information for wellness and psychological support were signposted around the department (Figure 1).

#### Patient and family liaison service

Restricted access to visitors was mandatory for safety. This placed a strain on family members unable to visit their relatives and consequently a patient liaison service was set up for a regular communication system. Doctors of various levels volunteered to call patient families and keep them informed. Charitable donations allowed some of this communication to be done via video calling using hand-held devices.

#### Staff testing

Testing for COVID-19 via nasopharyngeal swabbing was available for symptomatic staff in order to isolate if positive or facilitate a quick return to work if negative, and symptoms have resolved. Testing for staff who lived with symptomatic family members was also offered. Support was provided for ill staff at home, including organising testing during isolation and return to work interviews.

### Role of radiology

#### Radiology command structure

As the Covid-19 pandemic unfolded, the implications for imaging services quickly became apparent necessitating a novel response. In line with the national Emergency, Preparedness, Resilience and Response (EPRR) framework,^7^ the hospital quickly declared a major incident and initiated a three-tier command and control structure with Gold (strategic level), Silver (tactical) and Bronze (operational). The radiology department implemented its own “Bronze” and “Silver” command structure to streamline decision-making and ensure the 400 staff in the department were deployed appropriately to respond to clinical need.

The Radiology Silver command group comprised the Clinical Director, Deputy Clinical Director, a senior consultant/service lead, the General Manager and a representative from Radiology Bronze on rotation. From March, Silver command met daily via teleconference 7 days a week, and was supported by a Radiology Bronze tier, comprising four senior members of the operational management team (Superintendent Radiographers/Service Managers). The function of Radiology Bronze was to implement the decisions and overarching tactical plan that Radiology Silver had laid out. They worked on a shift system to ensure there was at least one member of staff onsite 7 days a week between 08:00 and 18:00. A standardized handover template was developed to communicate essential information between team members and ensure continuity of practice. The function of the Silver and Bronze command is summarised in Table 2.

**Table 2.** Summary of the Silver and Bronze team command functions

Command structure	
Silver command	Bronze **command**
Develop the strategic response to the incident informed by hospital directives and national policies	Ensuring radiographic staffing levels were sufficient to meet service demands on a day-to-day basis, rostering staff as necessary to increase or decrease capacity
Develop and implement a new model of service delivery based on 24/7 working	Monitoring PPE stocks to ensure that supplies were adequate
Monitor activity levels across the department, turnaround times and quality assure service delivery to ensure patients’ needs were being met	Acting as a point of escalation for urgent operational and Covid-19 issues
Ensure that the working environment was safe for staff and that they worked within their clinical competencies	Promoting staff welfare and wellbeing
Develop new staffing models for the radiologists so there were resident consultants on-site 24/7 with inbuilt cover for sickness	Monitoring staff sickness and coordinating testing of symptomatic staff
Develop a new radiographic staff model to cover core inpatient and urgent outpatient imaging across all modalities in hours with increased staffing out of hours	Liaising with the ED and other departments (*e.g.* theatres/wards) to maintain the flow of patients throughout the imaging department
Establish satellite radiology services at other sites to support the continued delivery of cancer services	Attending daily site/ED operational meetings
Oversee the redeployment of non-essential staff to support the wider hospital response to the pandemic	Communicating service changes and updates to clinical practice/PPE guidance/SOPs to the imaging department

ED, Emergency department; PPE, Personal protective equipment; SOPs, Standard operating procedures.

The command structure played an essential role in consistent communication and enabling the imaging department to react quickly and effectively to the pandemic. It provided a structure for staff at a time of great uncertainty and centralised decision-making, reduced duplication of tasks and effort, providing a clear route of escalation for problem-solving.

### Role of radiology in diagnosis

**Figure 2.**
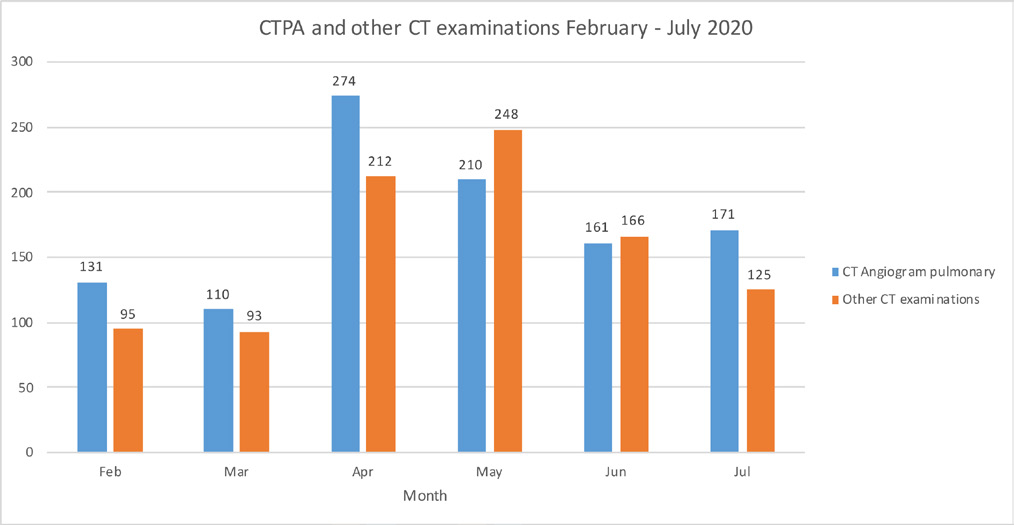
Bar chart of the Accident and Emergency and inpatient CT pulmonary angiogram and other CT chest examinations performed from February to July 2020. CTPA, CT pulmonary angiogram.

**Figure 3.**
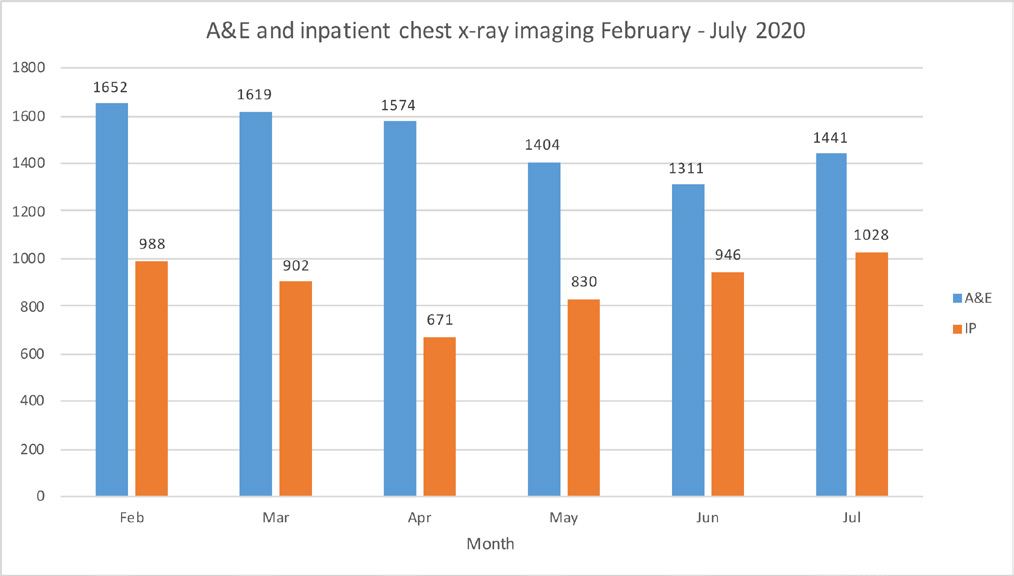
Bar chart of the Accident & Emergency and inpatient chest X-rays performed from February to July 2020.

While non-urgent imaging immediately reduced significantly, the demand for COVID-19-related CT pulmonary angiogram (CTPA) and other chest CT imaging increased (Figure 2). An large increase CTPA imaging was probably related to an increased prevalence of pulmonary thromboembolic disease in patients with COVID pneumonitis.^8^ A&E and inpatient chest X-ray demand remained high despite a large decrease in non-COVID-19-related A&E admissions (Figure 3). Chest radiography was used for screening symptomatic patients with suspected COVID-19 and could be interpreted immediately by the point-of-care clinician, but ideally a rapid report issued by a radiologist or reporting radiographer allowed for interpretation of subtle changes, avoiding delay in management and risk of a COVID-19 patient being sent to a non-COVID-19 ward. In other health facilities with high disease incidence, CT may have been used as a screening tool for the diagnosis of COVID-19, especially where there was limited access or delay to viral testing.^9^ We had local access to rapid viral testing, and used the chest X-ray as a tool to assess for lung changes characteristic for COVID-19.

In the United Kingdom, there was no nominated role for CT in the diagnostic assessment of suspected COVID-19 infection.^10,11^ Only patients presenting with abdominal symptoms and trauma requiring a CT abdomen, the addition of CT chest played a role in detecting the presence of possible COVID-19 infection, thus helping to stratify risk in those requiring emergency surgery.^12^ Routine CT chest was not indicated in the pre-op assessment for elective oncological surgery, for those who were asymptomatic, in isolation and had a negative RT-PCR test.^13,14^

**Figure 4.**
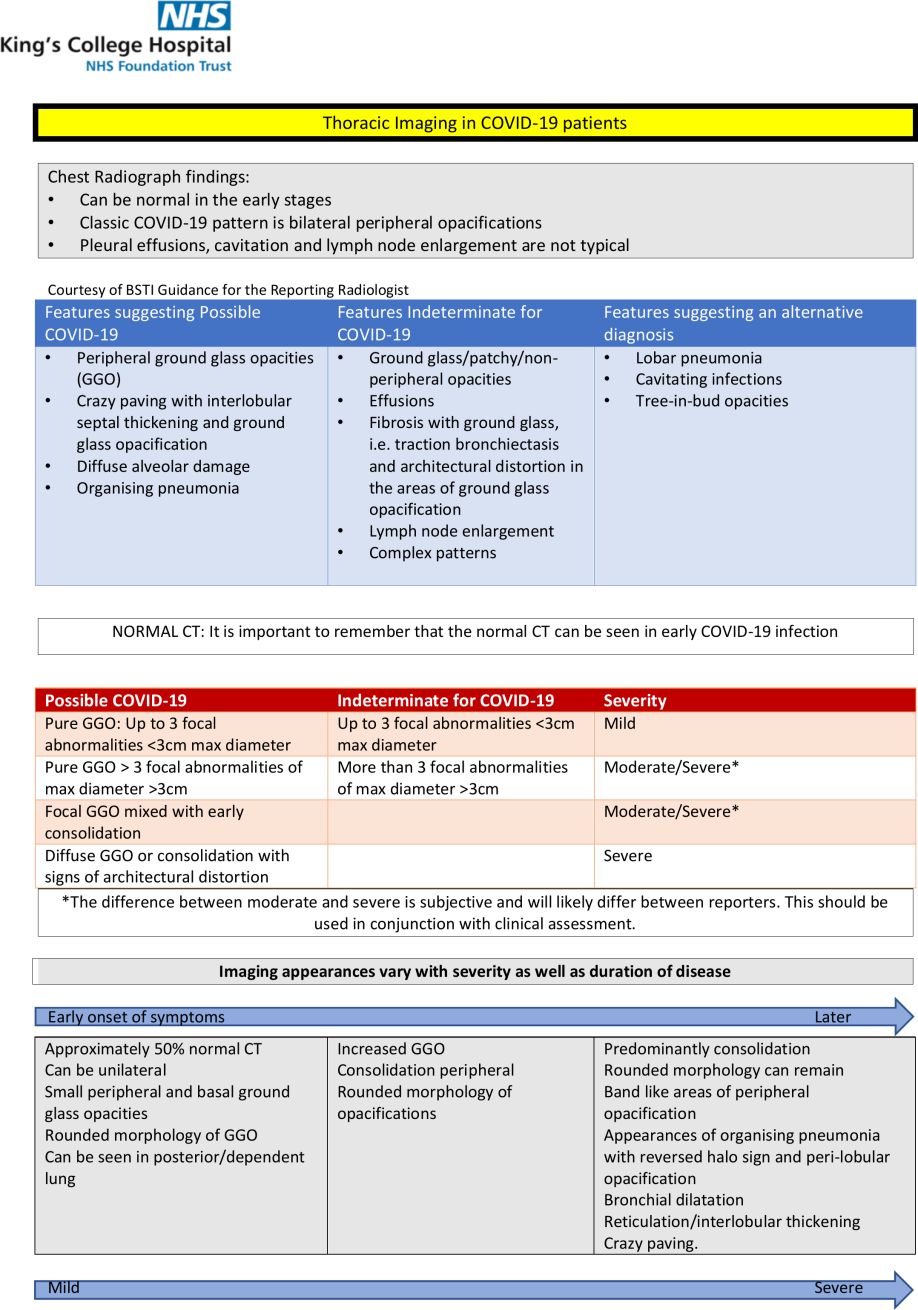
Departmental guidelines for the identification and reporting of COVID-19 on CT Chest. Courtesy of King’s College Hospital NHS Foundation Trust.

Departmental guidelines were produced and disseminated amongst radiology staff, for the identification and reporting of COVID-19 on chest radiography and CT. Reporting templates helped the reporter include important positives and negative findings that support or oppose the diagnosis of COVID-19, including severity index score to help prognostication (Figure 4).

### Operational modifications

Hospital-wide changes were made to improve patient flow, prevent the mixing of patients, and reduce cross-infection. Pre-COVID-19 imaging practice in our institution (King's College Hospital) was location-based, *e.g*. all central nervous system imaging was solely undertaken in the neuroradiology section of the department. In separating scanners to deal with either COVID-19 or non-COVID-19 patients, all scanning protocols needed to be available on all scanners; this was instituted across all scanners. Any “suspected” cases were treated as “COVID-19 until proven otherwise”, with the appropriate patient and staff PPE and infection control measures. The emergency imaging department was converted into a self-contained COVID-19 imaging department, including a CT scanner, two dedicated X-ray rooms and one ultrasound room that were in adjacent, and separate from the “clean” areas in the hospital. This left a number of plain X-ray rooms in the ED imaging unit available for non-infective patients, with a physical barrier was erected between the two areas. Signs indicated the single restricted entrance to this “COVID-19 dedicated” area. One MRI scanner and one interventional radiology suite were converted into dedicated COVID-19 areas, chosen as accessible with minimal exposure of infected patients to the department. All staff wore relevant PPE throughout their shift and each room was cleaned and decontaminated after each imaged patient. The department continued working in all other imaging modalities to facilitate the patients with urgent imaging requirements as well as those patients with possible cancer diagnosis or under active surveillance.

### Portable imaging

One of our key principles was to utilise portable imaging as much as possible to reduce the risk of contamination and cross-infection in the radiology department and whilst transporting the patient. All inpatient chest X-rays and ultrasound examinations were converted to portable procedures. Each ICU had a dedicated ultrasound machine, appropriately transducer equipped and portable digital X-ray machine, with wireless remote transfer of images possible. Portable imaging staff worked in teams of two to promote efficiency, reduce viral exposure, and prevent cross-infection between teams. Indication for the portable ultrasound imaging was strictly protocol-driven, and only for those patients that an ultrasound examination would change their management; liver, renal, venous thrombosis assessment were the most frequent requests. Bedside ultrasound imaging of the chest was undertaken by ICU staff as a point-of-care facility.

### Rapid deployment of home PACS workstations

To provide working capabilities from home with the possibility for isolation of essential personnel, 10 dedicated home reporting stations were set up and divided equally amongst radiology subspecialty consultants. Due to underlying hospital-based technical limitations, more than this number of home workstations could not be provided using existing infrastructure to allow enough stations with remote access and speed with sufficient quality to allow more home reporting stations.

### Spatial segregation

Interactions between different staff (clinicians, radiologists, radiographers, and sonographers) were kept to a minimum and social distancing was maintained. As much as possible, non-face to face communication was facilitated; referrals were undertaken solely by telephone. Preliminary reports in major trauma cases that previously would require in-person reporting were conducted using a remote workstation and results communicated by telephone. Of note, the major trauma patient numbers were significantly less during the government-imposed national population isolation. In order to ensure reliable network coverage for these, telephone Wi-Fi phones were procured and used across all teams in the hospital.

Outposts within the hospital were created for Radiology staff that was considered high risk and required self-isolation. Physical intra- and interdepartmental meetings, and multidisciplinary meetings were converted to virtual videoconferencing meetings. Where possible, non-essential physical meetings were cancelled. Effective methods of videoconferencing include Microsoft^®^ teams and Zoom^®^.

### Workforce modifications

Radiology staff was reconfigured to provide a 24 h onsite consultant-based service and the previously overnight outsourcing of imaging and any previous cross-site specialised imaging reporting ceased. Three consultant groups were created and rotated in a 3 week cycle. Each group had equal representation from each subspecialty including interventional radiology (IR), which allowed adequate subspecialist reporting and intervention performance. The registrar emergency rota followed a similar pattern. All medical staff employed as part-time was converted to a full-time working pattern with consideration of compensated remuneration.

Temporal segregation of the teams allowed adequate rest in between shifts to avoid fatigue, as well as prevent cross-infection of the teams in cases of spread of infection within another team.

### Redeployment

Many countries around the world that saw a peak in COVID-19 had difficulties in coping with the number of admissions and ventilated patients. Redeployment of doctors of all grades and specialties was needed to meet the increasing demand, particularly in the ICU, as well as the shortfall of doctors and allied healthcare staff from sickness-related absence.

Redeployment was managed within Radiology in a stepwise manner; dependent on the centralised command control of the hospital requirements for extra staff. The minimum support needed for safe acute imaging services was identified in order to allow the more junior radiology trainees to be redeployed and maintaining more experienced radiology trainees within the “team” of radiologists providing a 24 h service. The redeployment occurred in batches as the pandemic escalated with continually more senior groups were redeployed. Non-patient facing roles included patient family liaison roles, and radiology trainees provided additional skills to areas of redeployment such as central and peripheral line placement and onsite ultrasound. Where necessary, training was provided to the trainees prior to redeployment.

### Independent sector

There was a nationwide response led by the government to use all independently operated hospitals for treating non-COVID-19 NHS patients, with a particular focus on urgent and cancer work. This involved providing NHS care at London Bridge Hospital (HCA Healthcare UK), 3 miles North to the KCH Denmark hill site. In radiology, along with Guys and St Thomas’ NHS Foundation Trust and Barts Health NHS Trust, we provided diagnostic and interventional support for NHS patients, particularly post-operative acute complications in and out of normal working hours. Close cooperation was required between the Information Technology Departments of both hospitals to allow for the transfer of images and reports, allowing for off-site reporting. Any ultrasound examinations were performed by sonographers on a rota, and IR was supported from our department.

### Interventional radiology

The intervention suite was divided into COVID-19 and non-COVID-19 units to allow safe patient movement and infection control. There was a major shift in intervention services to COVID-19-related and emergency cases. The COVID-19-related procedures predominantly comprised of catheter drainages and central venous line placement. Specialist services such as the liver transplant programme continued but were reduced, as well as any associated liver intervention services. Oncology intervention services continued, in keeping with NHS directives to maintain cancer services.

The interventional radiologists and intravenous access teams helped the ICU teams with central venous and peripherally inserted lines and peritoneal dialysis line placement. Central venous line placement was performed at the patient’s bedside as much as possible. Tunnelled and complex venous lines were performed in the intervention suite.

Some operations were continued at the independent hospital, London Bridge Hospital (HCA Healthcare UK), such as transcatheter arterial valve implantation (TAVI). Two radiology interventionists, with separate complimentary skills, were available to provide emergency cover. Procedures performed were mainly catheter drainages, angiography and vascular access. IR cover for emergencies was also provided by the NHS radiology consultants to the Nightingale Hospitals.

### Nursing staff

Interventional radiology services switched from a 5- to 7-day service in preparation for COVID-19, requiring modification of the nursing rota to provide cover for the extended services. To facilitate social distancing, and continue urgent mainly cancer treatment, patient pre-assessment was converted to non-patient-facing via telephone assessment. Pre-assessment included screening questions for fever and other COVID-19-related symptoms. RT-PCR swab test for COVID-19 was not available for pre-assessment patients, therefore 14 day self-quarantine was required before the day of the procedure. Screening was performed on the day of the procedure by temperature testing and repeating the COVID-19 symptom questionnaire.

### Radiographers

The normal radiographer rota already provided a 24 h, 7 day service which was adequate to cope with increased demand for portable chest X-rays and CT requests. Rota modifications and training for adequate team skills mix were required for preparation. Overnight radiographers’ numbers were increased from 4 to 6 (equally distributed between CT, X-ray, and angiography) and weekend day staff from 9 to 17 radiographers to continue service in all areas. Portable X-ray requests increased from approximately 30 before the COVID-19 pandemic to over 100 requests per day during the peak of the pandemic. The rise in COVID-19 ICU admissions resulted in the significant increase in portable X-ray requests. Radiographers subspecialised in breast, nuclear and MR imaging had seen a marked decrease in service requirements due to the post-ponement of non-urgent imaging. As a consequence, all newly qualified X-ray radiographers and sonographers were required to obtain basic retraining to be redeployed to CT and for portable X-ray, to compensate for the rapid increase in workload.

### Sonographers

All non-urgent ultrasound examinations were cancelled, all primary care referrals and the previously established walk-in urgent examinations, *e.g*. testicular lump service continued. All patients with known COVID-19 were examined using a portable ultrasound machine at the patient bedside, and each ICU section maintained their own dedicated ultrasound scanners which had sufficient transducers for vascular, general and cardiac examinations. This helped avoid cross-infection and reduce the time needed for cleaning and decontamination between patients. Sonographers scanned in pairs; firstly, to assist one another with donning and doffing and secondly, teamwork helped improve efficiency and reduce fatigue.

A 7-day emergency rota was constructed with two teams and each day was separated into two shifts, with 3–4 sonographers in each team. Two sonographers were assigned to the London Bridge Hospital every morning to scan non-COVID-19 post-operative patients. Point of care lung ultrasound was performed by the point of care clinicians, using the dedicated ICU scanners.

### Breast screening

The South East London Breast Screening Service, part of the national NHS Breast screening programme, screens more than 70 000 females annually and was post-poned from 25 March onwards, allowing breast screening staff to be redeployed to support frontline services. All staff except one radiologist and two advanced practitioners were redeployed to assist in COVID-19 duties.

All outstanding mammograms were assessed and all patients identified with an abnormality were scheduled for a clinic. Those vulnerable and with equivocal abnormality were informed and given advice to monitor for breast lumps. Patients with suspicious findings were asked to attend the clinic. Symptomatic breast clinics continued, with face-to-face clinics converted to remote surgical consultation, followed if needed with limited one-stop service.

Return from redeployment coincided with 2-week-wait referral clinics, before restarting the screening programme. A priority list was produced of high-risk patients (*e.g.* positive family history) for breast screening recommencement, with all cancellations reinstated, with 22 000 patients affected. To increase capacity, extended days and weekends have been implemented for staff to handle the backlog.

### Nuclear medicine

Regular guidance was issued by the British Nuclear Medicine Society, including workforce modifications, triage and departmental infection control compliance. All non-urgent elective scan appointments were cancelled, including dual-energy X-ray absorptiometry (DXA), non-oncological positron emission tomography (PET) and single-photon emission computed tomography (SPECT), and non-oncological therapies. Oncological PET and therapies for malignancy were continued as normal at a 50% reduction, predominantly from patient anxiety of potential hospital exposure to COVID-19. A single facility for PET/CT did not allow the provision of COVID-19 and non-COVID-19 scanners therefore cleaning protocols were in place to reduce the risk of cross-infection. Patient appointments were staggered throughout the day and the waiting room was altered to reduce the number present within the department to allow adequate social distancing.

### Administration team

The office managers were responsible for maintaining and restructuring the administration service, as per trust guidance and instructions. Regular communication was maintained with senior management to facilitate regular operational changes due to the rapid rise of COVID-19 patients. Working hours were extended to 6:00 pm and the team worked on a rotational basis. Initially, there was “all hands-on deck” to post-pone or cancel all non-urgent radiology services and telephoning patients. There was redeployment of administrative staff to wards for caring roles and assistance when required.

### Training and education

A standardised approach was required for all radiologists in the interpretation of this new manifestation of a disease pattern. A uniform departmental reporting process was instituted to allow for the detection, assessment of severity and follow-up imaging for the COVID-19 patients. A large resource of online material sprung up worldwide, and these online webinars were effective for presenting the most up to date knowledge regarding the imaging and management of COVID-19. The decrease in subspecialty work and redeployment meant the post-ponement of formal training rotations nationwide, included both radiologists and radiographers. Despite this, learning could continue within the department utilising many platforms such as online resources and videoconferencing. Consultants and trainees prepared and carried out teaching presentations using online communication systems (Zoom and Microsoft teams). All research projects involving patient recruitment outside of COVID-19 studies were stopped. The radiology department switched attention to identifying imaging themes and actively managed studies of COVID-19 patients successfully.^8,15–17^

### Departmental wellness and psychological support

Within the radiology department, the formation of working teams created a sense of comradery. The skills mix within teams brought colleagues that would not normally work in a clinical setting, closer together, with a common goal of providing effective services during the COVID-19 pandemic. Communication between team members via social networks (WhatsApp^®^ messenger) proved effective for individuals to share experiences, voice concerns or to ask for help with challenging situations. Home baking with regular cookies and cakes brought a sense of comfort and togetherness.

The training programme director held weekly videoconferencing meetings with all trainees, along with all educational supervisors. Trainees, particularly those redeployed were encouraged to share their experiences and raise any concerns regarding working outside their comfort zones. The redeployed trainees acted as ambassadors for radiology, conducting teaching sessions on aspects of radiology related to the COVID-19 pandemic, via weblinks, tailored to ICU staff.

### Issues encountered

1. The Emergency Department Major Trauma CT scanner was used as the dedicated area for COVID-19 imaging, which also houses X-ray rooms and an ultrasound room. All major trauma had to be diverted to a more remote first floor CT suite resulting in slight scanning delays. Severe or life-threatening trauma patients were transferred to the COVID-19 converted emergency department scanner, overriding the designated status to avoid delay. The level and intensity of cases of major trauma dropped significantly, and only twice over the period was this COVID-19 scanner used for immediate life-threatening trauma cases.
2. Radiographers were initially overwhelmed and struggled to cope with the amount of departmental and portable radiography requests, but this improved with a revised provision to increase staffing and bolster staff at “pinch” points. This was achieved by increasing overnight and weekend staff; training and redeployment of junior radiographers, and radiographers from other modalities (nuclear medicine, MR and breast imaging) to meet the increased demand.
3. Not all multidisciplinary meetings continued with the same volume but due to reduced consultant availability “in-hours” when converted to a 24 h emergency rota and social distancing, a minority of tertiary specialties including hepatobiliary, neurosurgery and trauma could not access dedicated multidisciplinary meetings. Certain cases were still discussed as required on an *ad-hoc* basis. The scale of imaging cancellation was apparent despite urgent and cancer patients being offered imaging; many did not attend the hospital.
4. A breakdown of communication between patient, clinical teams and imaging services was apparent and required constant management. The common theme was that Radiology was not considered a front-line service. To manage this, the department ensured equal representation at management/COVID-19 meetings.
5. There were inadequate facilities for comfort or rest within the radiology department, and radiology was overlooked in regards to rest areas and charitable food contributions. Local perception of the actual daily work practice of a radiology department, and the patient-facing role, was significantly undervalued by the hospital’s non-clinical and administrative staff. This had a demoralising effect of the more junior staff members, and in particular the denial of access to donated meals overnight was deemed the most difficult to comprehend.
6. The ultrasound department is housed in a historically inadequate facility and the ability to delineate separate pathways was challenging. This was overcome by converting a room in the COVID-19 area to an ultrasound room and performing portable ultrasound examinations.
7. The speed and scale of the appropriation of the independent sector were not adequately communicated to the Radiology Department, as hospital administrators lacked a clear understanding of the imaging support needed. Radiology was excluded from decisions surrounding the types of surgery performed. The inability to provide an IR service for peri- and post-operative complications became evident very quickly. Concerted efforts to manage this situation where needed.
8. All backlog reporting in the department was cleared during the period of the COVID-19 pandemic, in a matter of 2–3 weeks. The report availability with ongoing imaging was very timely.

## De-escalation

Sustainability of the radiology department in the long-term is important in a prolonged pandemic, especially with the ever-present risk of a second wave of COVID-19 admissions with the lifting of restrictions in public places. De-escalation is a transition to pre-COVID-19 services by means of a gradual reintroduction of non-urgent imaging and intervention services. A timeline of escalation and de-escalation steps taken is summarised in Table 3.

**Table 3.** Timeline of escalation and de-escalation steps taken

Timeline of escalation and de-escalation steps taken	
Dates	Steps
17 November 2019	First case Hubei Province China
29 January 2020	First case in the United Kingdom (York)
End of February	First COVID-19 diagnosis at Kings College Hospital
11 March	WHO categorise the infection as a pandemic. Trust initiates major incident framework. Command structure operational to manage response to COVID.
12 March	All elective activity paused with the exception of surgery for cancer and other life-threatening conditions. Coronavirus pathways and radiology operational modifications initiated.
17 March	Restricted visiting policy implemented
23 March	Directives received by Medical Director via NHS England and NHS Improvement for 24 h consultant rota. Staff well-being hubs setup within the hospital.
End of March	24 h rota implemented in Radiology. Home reporting stations installed.
2 April	Start of radiology trainee redeployment
3 April	NHS Nightingale opened
9 April	UK peak of COVID-19 deaths
Mid-May	Acute phase of COVID passed. De-escalation initiated. Return to pre-pandemic working patterns.
1 June	Staff working at home start to return to work
19 June	Restart of elective services
End of June	Return of redeployed trainees
1 August	Shielded staff return to work

COVID-19 in-patients and new admissions had significantly subsided and de-escalation measures were initiated by mid-May. A step-down from emergency 24/7 cover with the reintroduction of the overnight outsourcing was introduced to increase consultant presence during normal working hours, and to handle the increasing workload from returning services. Staff returned to their original roles within the radiology department, rota coverage was reduced. Staff vacation leave was reintroduced, and staff encouraged to take this time.

The introduction of mobile units for CT and MR scanners was rapidly instituted to create more capacity to handle the backlog of imaging requests. Maintaining infection control, social distancing and continued use of appropriate PPE was reinforced. Segregation of inpatient and outpatients, and zoning of the hospital; clean areas *vs* COVID-19 scanners, was rapidly put in place.

The Radiology command structure was revised accordingly, which enabled the operational team to return to their normal roles and focus on restarting elective services. The radiology silver and bronze “crisis” teams were replaced by a comprehensive “reset and recovery” team, tasked with the reinstitution of patient imaging services.

A staff antibody testing programme was set up and made available to all clinical areas and office administrative staff. Front-line staff and those deemed as high risk were prioritised. The programme formed part of a national initiative led by NHS England, identifying specific antibodies, SARS-COV-2 IgG, with a positive test indicating previous infection with COVID-19. The results will provide more detailed knowledge about the prevalence of COVID-19 across the country, and to better understand how the disease spreads.^18^

Radiography students remained offsite with reintroduction planned for September. Formal radiology trainee curriculum and training were reintroduced. Post-redeployment interviews were carried out, including continuing educational supervisor support to address trainees concerns and identify individuals’ training needs and those requiring extra psychological support. Health risk re-assessments were performed to aid a safe return from shielding. Those who required to continue isolation were given academic opportunities to contribute to research projects, audits, quality improvement projects, information collection for articles, and education.

### Planning ahead

Working in these challenging conditions identified new working patterns which also brought benefits in direct patient care and working within and between teams. The positive learning/outcomes need to be recognised and continued into the recovery phase of the “new normal” working pattern. Virtual clinics and telephone consultations have proven effective in delivering non-patient-facing care during these times and may offer some benefit post-pandemic for patients. Post-initial response reflection should optimise standard operating procedures which should be in a state of readiness for future outbreaks. The introduction of virtual MDMs was vital for the continuation of this service during the pandemic but will also benefit practice going forward. This reduces numbers in a room for social distancing but also removes the need for traveling between hospital sites. Installing home reporting stations is also crucial for increasing the number of diagnostic radiologists able to work remotely from home. Education and training have continued to be done remotely on videoconferencing platforms, which allows greater access for those who work offsite or from home (less-than-full-time trainees). Good interdepartmental relationships developed between redeployed trainees and intensive care. A weekly registrar led multidisciplinary/education meeting was set up for the intensive care unit to discuss important cases and imaging findings, which has continued.

There are certain aspects that would be done differently if an escalation response is again required. In retrospect, there were more resident on-call consultants overnight than required to cope with the increased COVID-19 imaging and intervention workload. In future, reducing the consultant overnight rota and continuing the third-party outsourcing teleradiology company services would be considered, especially if elective work is to be continued.

The reorganisation of the department in preparation for a pandemic is not unique to radiology and certain aspects can be implemented across different departments and specialties. These include, restructuring patient pathways to avoid cross-contamination; implementation of a 24 h subspecialist consultant rota, if not already in place; providing wellness and psychological support for staff; converting physical meetings to videoconferencing and virtual meetings where possible to facilitate social distancing; continued training and education via electronic resources and virtual teaching sessions.

## Conclusion

The pandemic has proved the resilience of many hospitals around the United Kingdom and their ability to adapt in short notice and rapidly changing policy and procedures. We describe a process of escalation and de-escalation, to adapt to the challenges of an outbreak and ensure excellent patient care, staff well-being and business continuity. Emphasis is made on infection control, the flexibility of staff and departments, and teamwork. We hope that the KCH response to the COVID-19 pandemic will act as a guide for other radiology departments in preparation for future outbreaks. The essential nature of the imaging service is reinforced by the crucial role the imaging department played in the management of this pandemic.
